# "Who am I? Where am I?" Experiences of married young women in a slum in Islamabad, Pakistan

**DOI:** 10.1186/1471-2458-9-265

**Published:** 2009-07-28

**Authors:** Saima Hamid, Eva Johansson, Birgitta Rubenson

**Affiliations:** 1 Global Health, Department of Public Health Sciences, Karolinska Institutet, Stockholm, Sweden; 2 Health Services Academy, Islamabad, Pakistan; 3 Nordic School of Public Health, Gothenburg, Sweden

## Abstract

**Background:**

In Pakistan, 16% of the women aged 15–19 years are married. Many get married shortly after they attain menarche. This study explores the preparedness for and actual experiences of married life (inter-spousal relationship, sexual activity and pregnancy) among adolescent women.

**Methods:**

Among married adolescent women residing in a slum of Islamabad ten were selected with the help of a community health worker and interviewed qualitatively till saturation was reached. They were interviewed three times at different occasions. Narrative structuring was used to explore how the participants represented their background, social situation, decision making and spousal communication and how they explained, understood and managed married life and bore children.

**Results:**

Two categories identifying the respondents as either submissive-accepting or submissive-victims emerged. The married young women who belonged to the accepting group lived under compromised conditions but described themselves as satisfied with their situation. They were older than the other group identifying themselves as victims. However, none of the respondents felt prepared for marriage. Women belonging to the victimized group experienced physical and verbal abuse for their inability to cope with the duties of a wife, caretaker of the home and bearer of children. Their situation was compounded by the power dynamics within the household.

**Conclusion:**

Knowledge about sexuality could prepare them better for the future life and give them more control of their fertility. Adolescent development and life skills education need to be addressed at a national level. There is need for innovative interventions to reach out and provide support to young women in disadvantaged homes.

## Background

One in six adolescents aged 15–19 years is married in Pakistan [1]. Marriage takes place shortly after menarche. Women, who marry, move from their familiar home to the home of the husband. They initiate sexual activity with a husband they barely know, and soon become mothers. Many find themselves socially isolated and with poor inter-spousal communication [2,3]. Adolescent marriages lead to early pregnancies and complications due to immaturity contributing to their vulnerability [4].

In the Pakistani context, importance is given to preserving the chastity of young women before marriage. Their sexuality is tightly controlled by their guardians [5]. Seclusion norms (*purdah*) are common from puberty onwards and unmarried women have restricted mobility [6]. As discussions of sexuality are discouraged little is known about sexual attitudes and behaviour of young people in Pakistan [5]. Many young women are poorly informed about sexual issues, reproductive biology and health [7,8]. Low levels of school attendance, lack of sex education and norms that prohibit discussion of sexual issues preserve ignorance [6,9,10]. Men are relatively more knowledgeable about puberty, pregnancy, family planning and sexually transmitted infections [11].

Adolescence is a period of significant change and transition towards social and economic independence. Boys and girls make important choices and build critical capacities during this period [12-14]. Entering into marriage adds to the complexity of the situation for young women in Pakistan [15]. History and environment jointly influence the value systems and norms of a society, which include settlement patterns, household structure and division of labour. Aspects of adult personality are culturally expressed and projected in rituals and belief systems e.g. reflected in women's limited control over their own lives [16,17]. Men have access to a variety of information sources outside home while young women are restricted to a limited number [15]. Gaining information is event based, whereby specific events e.g. puberty and marriage trigger information provision to young people, however often too late to be educative.

Limited research is available on factors that shape the married adolescent women's reproductive behaviours and experiences. A study conducted in Bangladesh [18] on married adolescent women showed that many had borne children before they were emotionally and physically ready. Poverty, economic conditions, marital insecurity, politics in the household, absence of dowry and rivalry among family, co-wives and in-laws made these young women comply to decisions made by others in order to survive. They had been compelled to bear children or terminate them subject to the decisions of others in the household.

The objective of this study is to explore how young women (13–19 years) are prepared for married life. It explores their experiences and knowledge of married life in relation to sexual activity, child birth and inter-spousal relationship.

## Methods

A qualitative approach was used to explore experiences related to married life and to examine the relationships between different social levels inspired by Bronfenbrenner's ecological framework [19]. According to Bronfenbrenner, individuals are nested in environments or systems (terms used interchangeably), each of which is contained in the other, from more proximate to more distal determinants. All of the systems are interdependent and interactive, with changes in one system affecting the other systems. This human ecological framework permits examination of linkages between levels of environmental context. This ecological approach relates changes in the individual to the social and physical environment [19].

The research team comprised of a Pakistani medical doctor with a public health focus (PI), a Swedish public health expert, a Swedish nurse with a focus on childhood transition and rights, and a Pakistani community worker as a gate keeper. This broad composition of the research team brought different perspectives to the study. The community worker played a key role in giving the emic perspective to the study. She met women residing in the community regularly and was an advisor for health and social issues.

She shared her insight of the culture and norms of the community under study with the PI. The interviewing was done by the PI followed by data interpretation and analysis together with the rest of the research team members. The outsider's view of the Swedish scientists broadened and enriched the understanding of the material.

### Setting

The study was conducted in a slum community in the outskirts of Islamabad city. There were 900 houses in the community and almost 400 households had more than one family living in one house. Most of the residents were labourers working on daily wages. Most residents were illiterate belonging to the low socio-economic group with poor hygiene and nutritional status. They belonged to different ethnic groups in-migrating from different areas of Pakistan. Living together they had adjusted to each other and developed a common culture. The women had usually not attended school beyond primary level and many had never even begun. Shortly after puberty they were married and moved to live with the in-laws for cultural and financial reasons. Most women had limited opportunities for employment and education. There was one sewing school run by an NGO where those who could afford attended classes. Going outside of the house required an elder from the house to accompany them.

### Participants and Data Collection

Through purposive sampling married adolescent women who were willing to share their experiences were approached by the community worker. If one refused, another participant fulfilling the criteria was included in the sample. Thus ten participants were interviewed by the PI. Multiple meetings with the participants were held so that the researcher developed rapport with the participants. The interviews were conducted in private, with no family member or the community worker around. This helped break the ice and gave the participants a chance to re-think past events and remember other associated experiences that could be shared at subsequent meetings. Unstructured qualitative interviews [20] were chosen as the appropriate method.

A married life calendar was developed to explore the experiences of the participants by discussing their needs at the time of marriage, their access to information, source of information prior to marriage and the experience of sexual activity, communication with their husbands and childbirth.

The interviews were tape recorded with the participants' consent and transcribed within two days by the PI. The researcher went back with the written narration for verification followed by any further probing identified as needed by the research team.

### Data Analysis

The complete transcripts were read several times to gain a general sense of the experiences of the participants. Bronfenbrenner's model was kept in mind when exploring how the different systems affected the young women and what the linkages were between them. The interviews were compared and themes and events searched for which could explain the experiences of the women and the way they identified themselves. Narrative structuring was used to demonstrate how the participants described their background, social situation, decision making, spousal communication and how they explained, understood and managed married life and bore children. Narratives are not the exact records of what actually happened [21]. The historical truth of the participants' account is not the primary issue. Narrativisation is the point of view of the individual based on his/her experiences and interpretation [21]. The same event is narrated differently depending on the values and interests of the narrator and to whom the story is told. As the past is selectively reconstructed troubling events will be recalled and narrated differently. Researchers have to assess whether the account of the events are persuasive and plausible [21]. Through the process of "narrative finding and narrative creating" [20] two main thematic narratives were developed depicting the two main identities found in the stories of the participating women.

The perspectives of the different researchers in the team enhanced the trustworthiness. Combining the insider and outsider perspective contributed to the understanding of the material. The findings were discussed with the community worker for verification.

### Ethical Considerations

Ethical clearance for the study was sought from the national research body (Pakistan Medical Research Council) and from the ethical committee of Karolinska Institutet, the medical university of Stockholm in Sweden. Before starting the interviews the study was introduced to the community. Verbal consent from all the participants was obtained prior to their participation in the study. The study was explained and queries addressed before the start of the interviewing. The consent of the participants was obtained after the permission from the decision maker in the house had been given. In most cases it was the mother-in-law in the absence of the husband. The consent statement was read out clearly explaining the study objectives and the expectations on the study participants to facilitate their understanding. Study participants were assured of confidentiality. The study could raise reactions and emotions difficult for the participants to handle and the community worker was asked to be available for those who needed extra support and comforting and to facilitate referral through the NGO if needed.

## Results

The participants with their individual socio-demographic characteristics are presented in Table 1. Major topics reflective of the personal narratives include the following categories: (a) family network and its influences on decision regarding marriage, (b) puberty and menstruation, (c) preparation for marriage (dowry, relationship with the new family members), (d) sexual activity (e) contraception and child-birth (f) role of the husband and other family members, (g) future aspirations and expectations.

**Table 1:** Profile of Young Married Women

**No**	**Age**	**Marriage age**	**Information about menstruation**	**Decision about marriage**	**Husband's characteristics**	**Living arrangement/s**
1	19	18	**Friend **informed on menstruation	Parents	25 years, 8 years schooling, shopkeeper	In parental home **(narrative 1)**
2	19	17	**Mother **taught practicalities	Uncle	20 years, 10 years schooling, employee	With in-laws, parents in same city **(narrative 1)**
3	20	19	**Friend **taught practicalities	Elders of the family	22 years, 10 years schooling, painter	With in-laws, parents in the village **(narrative 1)**
4	20	18	**Mother **taught practicalities	Parents	21 years, 8 years schooling painter	With in-laws, parents in next street **(narrative 1)**
5	17	16	**Teacher **taught menstruation and hygiene	Parents	24 years, master student	In parental home (**narrative 1)**
6	13	13	**Mother **taught practicalities	Mother	15 years, no school, labourer	With in-laws, parents in the village **(narrative 1)**
7	18	17	**Grandmother and aunt **taught practicalities	Father and Aunt	20 years, 5 years schooling, taxi driver	With in-laws, parents in same city **(narrative 2)**
8	15	13	**Sister **taught practicalities	Parents and Uncle	18 years, 10 years schooling, farmer	In parental home **(narrative 2)**
9	13	14	**Aunt **taught practicalities, managed alone first time	Father and Stepmother	30 years, 10 years schooling, tailor	With in-laws, parents in same city **(narrative 2)**
10	17	13	**Friend **taught practicalities	Father	25 years, no school, shopkeeper	With siblings in parental home **(narrative 2)**

The following section is based on the stories of all the participants from which two thematic narratives were developed. They include selected verbatim quotes from some of the participants that illustrate experiences common to them. Themes helped to focus on the different parts or facets of the stories and served to compare participants' stories and illustrate the similarities and differences. Through deliberations and discussions the participants could be sorted into the following two groups: the submissive-accepting and the submissive-victims. Pseudonyms are used to protect the identities of the participants.

All the participants expressed their need for better preparation before marriage. The young adolescent women viewed marriage as the end of their childhood and were overwhelmed by the responsibilities that they were expected to fulfil after marriage. They were not prepared for initiation of sexual activity. None of the participants knew that sexual activity leads to conception.

### NARRATIVE 1

#### Submissive and Accepting

My name is Shakira (Thankful) and I am 20 years old. I got married two years ago to my first cousin who is twenty four years old. He is a painter and has completed ten years at school. We live in a joint family system with my parents-in-law and my husband's married brothers and their families. I attended school for five years but when my father could not take me to school I dropped out.

I was engaged at birth and wore a string around my wrist. My aunt would refer to me as hers but I never understood why. Before my "nikkah" (legal marriage) at 14 years of age I menstruated two to three times. I had learnt about it in school and since I was close to my mother, she told me that it was normal and I was to have them monthly for five to seven days. She said I was no longer a child and should cover myself properly. She told me I had to be responsible as I was going to be married soon. I did not talk to my husband before marriage.

No one prepared me for the physical relationship I was to have with my husband. After three days of marriage I guess someone must have talked to him. That night he gave me 1000 rupees. He said that we had to start life together. I understood what he wanted, a woman knows. You age overnight the day you are labelled married. I was shocked by my experience, felt as if something bad had happened. My husband felt guilty for giving me pain. The next day I expressed my shock to my mother. She simply replied, "This happens!" It took me 15 days to settle down. I had my periods once after marriage and then I conceived. It would have been easier if my mother had prepared me for the physical relationship with my husband. I had married friends who talked but I never really understood the meaning of marriage. I did not know that sexual activity would lead to conception. I missed my periods and mentioned it to my sister-in-law who told my mother-in-law and my mother. Both were thrilled. Seeing my confusion my mother told me that when I was born she had the same *problem *implying that I was going to have a baby. I was upset as I felt that this was too soon and I was too young to have a baby.

My pregnancy went fine. The doctor came to deliver the baby at home. The baby was exclusively breast-fed and vaccinated. My mother came to help me after my delivery as I was weak. She taught me everything. I couldn't have managed without my mother's help. She would assist me in the household work. The baby was one year old when I had my menses once. I did not know that I was pregnant till my older sister-in-law asked me to have the pregnancy test done again. A few days later I had spotting and was taken to the doctor who told me that I had miscarried.

My husband has gone to Middle East as a labourer for three years. He sends money to my father-in-law who gives me some for my expenses. I like the joint family system. My parents-in-law love me and I attend to the household chores with my sister-in-law.

My childhood is gone. I have entered into a responsible life. If I have a daughter, I will marry her at 20–25 years after she has completed her studies. My husband says we should have no more than two to three children. He has seen his brother with his five children. My husband believes in educating the children. We want to be financially independent.

### NARRATIVE 2

#### Submissive and Victim

I am Masooma (Innocent) and I am 15 years old. I always wanted to study and my mother encouraged me. My life changed when my mother died three years ago. She burnt herself to death after having a fight with my father. He pretended to be asleep while my mother poured kerosene oil on herself and set herself on fire. My six year old brother watched and cried. She died three days later. Our father could not look after us. I did not know how to attend to household work or cook. My mother always had told me to concentrate on my school work. For almost a year we struggled like this. When I had my first periods I thought I was going to die. I was the oldest amongst my siblings. I have four younger brothers. I told my father I had urinary infection and he got me some medicine. When my aunt came to visit us three days later she told me that this is part of growing up.

One year later seeing the pathetic state of the house and children my father re-married. We were very upset and did not like our stepmother. She told my father to wed me off and in less than a year's time I was out of the house. I came to know of my marriage only one week before my marriage. I was going to school and was in class eight. I wanted to study and not leave home but my father blackmailed me into it by saying that he might die and that boys can live on their own but women need security. I therefore obliged him by agreeing to my marriage.

I found out that my husband worked as a clerk in an office and was 16 years older than myself. I cried a lot but look what happened. I am five months pregnant. My parents-in-law want a grandson as their other son who is married has four daughters. My husband's two sisters and one older brother are married too. His younger brother is getting married next year to his cousin. My mother-in-law took me to the hospital as I was vomiting a lot at home. When I saw the nurse and the doctor scolding the patients I was scared. I found that very insulting. I am afraid to go there for my delivery. My mother-in-law says home deliveries are convenient and comfortable.

My husband wants his things his way, wants the house to be in order, parents happy and having no complaints about me. If he gets upset he starts beating me. I hope I have a son so that I am respected.

When I got married I did not think that I would have a life like mine. My stepmother is far better than my mother-in-law. I am responsible for all household work. At first when I would mess up my work or cooking, my mother-in-law would get upset and hit me. She would instigate my husband and he would beat me up, too. Hit me with whatever came into his hand. My mother-in-law likes to create a drama.... I complained to my father but my husband poses to be so sweet in front of him that my own father does not believe me. On our wedding night he really freaked me out. I was barely 14 years old. He gave me a lot of pain. I was so upset. I would not let him touch me. I resisted for a couple of months. He complained to his mother who told him to tie me up and have sexual activity with me anyway. She said that I was very cunning and not a child. My husband complained to my father. My father and the community health worker came over and talked to me. I felt cornered and I let nature take its toll. I still do not find my physical relationship with my husband pleasant. Once I conceived I thought my husband would let me be. I thought the whole idea was to conceive, which I did but it still continues. I did not know initially that such relationship leads to childbirth but I learned this through the community health worker. I thought having a child would change things in my life. I wonder if it really will!

There is constant nagging at home. My mother-in-law says I am not innocent and my husband says that I have to learn to be obedient and submissive. I am not allowed to leave home unaccompanied. I worry for myself. Where is my home and who am I?

## Discussion

The two narratives based on the interviews of the study explain how the different levels of family, community and society impact on the young women's lives. The parents by deciding about their marriage often without their involvement and consent, the parents-in-law by often taking on an oppressive and demanding role and the society with its expectation that young women be obedient, all contribute to their submissive nature.

### Home Situation and Preparation for Marriage

The interviews showed that the position of young married women is very vulnerable in the low-income setting of the study. At the age of 13–19 years they pass from being a daughter to a daughter-in-law and wife in a new home. The limited information they had about marriage did not prepare them for what was waiting. They were raised to be obedient and not question decisions of the elders. They were taught how to adjust in their new homes but received no or deficient information on or around sexuality from their mother or other members at home. Women belonging to the first group came from stable homes where parents were keen and supportive in the settlement of their daughters in new homes. This support continued after marriage. Two respondents continued to live with their parents after marriage and were provided facilities to help secure a better future. The women belonging to the first group living with the in-laws were welcomed and continued to have support of both their parents and in-laws after marriage. If the parents lived nearby these young women felt more at ease in their new homes.

The women identifying themselves as victims came from families where they had felt unwanted, often with a stepmother replacing the dead mother. Three out of four respondents came from households where the young women were exploited and in a disadvantaged situation even before marriage. This feeling of not being wanted followed them into the new home. They were married in families where they did not feel welcomed, had low status and were victimized. The findings suggest that there was a lack of interest by the parents and guardians in finding a good home for these women. Marriage was in some cases perceived as putting the responsibility of these young women on others.

The traditional way of preparation when the girl is mentally prepared to move into a new home and environment worked to some extent for the first group but failed for the second, who apparently had little preparation, which can explain their discomfort and unhappiness. The move to a new environment worked for the first group as long as the expectations in the new home coincided with the preparation before the marriage.

The role of the husband was closely tied to the family system in both the first and the second group of respondents. His attitude towards his wife was consistent with the level of welcome that she received in her new home.

### Adjusting to the New Situation

The women in the study had moved into a life situation which in many ways was foreign to them. They all struggled to communicate with their husbands and other members of the new families. All women were surprised or even shocked to experience sexuality. They had either received no or deficient information on the topic prior to their marriage. Even the upcoming marriage did not cause any discussion on or around sexuality with their mother or other members at home.

Despite not having been prepared for marriage, the women accepted the situation and adjusted to their new environment as seen by the participants represented by the first narrative. However, others were unable to adjust and hence were struggling and suffering in their new homes, represented in the narrative of the victims. Acceptance is here defined as the participant's adjustment to the environment and was determined by her own experience of marriage, a comparison with others in worse situations and the expectations she had in accordance with what her mother had told her and prepared her for.

A review of the two representations identified in the life-stories of the young women showed two distinct modes of reacting to and coping with the new challenges. In the accepting narrative the young women felt welcomed and enjoyed their new status and the husbands' families showed preparedness to receive and welcome them. The accepting women were older than the women identifying themselves as victims, and they were able to undertake and carry out the expected household chores. They were also communicating with their husbands and were prepared to take the new role and avoided conflicts in the new home. They were in a position to plan for the future and looked forward to it as opposed to the second group where the young women saw themselves as the most inferior in their new home with no hope for the future. They were also expected to take on the role of a wife, a daughter-in-law and a mother with little support from their new families and lacking the support of the old family. This often led to conflicts as they were unable to cope with their responsibilities and interpreted this as being unprepared for their new role. They told about being physically and verbally abused for their inability to cope with their duties but saw this as normal based on earlier experiences and what they had heard about married life. The power dynamics in the extended household were clearly demonstrated in their life stories. These findings are similar to those brought out by other researchers both in Pakistan [10,15,17] and in the Bangladesh [18]. These participants had no dreams for future. The disadvantages in childhood by belonging to dysfunctional families continued into adulthood and marriage. The findings suggest that dysfunctional families marry their daughters into dysfunctional families and supportive families select equally supportive families. This is supported by Mahmood [2003] who suggests that the prevalence of family dysfunction is underestimated in Pakistan. He found verbal abuse, physical abuse and social pressures amongst 30–40 year old married women living in joint families prevalent across all socio-economic strata; such patients with dysfunctional relations often presented to family clinics [22]. Another study in urban areas of Pakistan [23] exploring the importance of social relations and depression among pregnant women showed that poor social relations with husband, in-laws, household work and pregnancy symptoms predicted depression.

Certain similarities between the two type narratives were identified. The mother-in-law or the head of the household was expecting a pregnancy and taking all decisions in relation to health care needs. The women had no financial or social independence nor were they in a position to decide on purchasing things they needed. These findings are similar to findings in other studies [3]. Although all would have liked to learn more about sexuality before marriage they had not dared to ask for clarity on the deficient information. Delaying the first pregnancy was desired by the young women but due to lack of knowledge and inability to speak with their husbands no contraception was used. Similar to other studies most men were embarrassed even to talk to their wives in the presence of other household members [15] and the wife was expected to find her place in the new home by pleasing everyone. The couples had little time together and the young women soon entered motherhood. Reproductive health and rights stipulate women's autonomy over their bodies and lives including the right to decide when to bear children [24]. For the women in the study conception was a possibility to gain status, fill an emotional void and at the same time the pregnancy was a surprise and a shock as they were not aware of the connection between sexual life and conception. The young women accepted decisions of others regarding their marriage and subsequent child bearing which was concurrent with their submissive nature.

### Methodological Considerations

To ensure trustworthiness the findings were shared with the community worker who recognized them as an adequate representation of the participants' lives. The respondents were interviewed multiple times, which added to the richness and credibility of the data, as the participants could expand on and explain earlier responses. The diversity of the research team members enriched the analysis and their different perspectives helped tease out the narratives and their meanings. The findings of the study cannot be generalised to young women in Pakistan, but the richness of the data suggests explanations for the vulnerability and difficulties many poor, young women in Pakistan experience.

## Conclusion

Our study of young women shows that they were poorly prepared for marriage. Whether young or old, whether accepting or victimized all the participants lacked access to correct information regarding sexual activity and its consequences at or around the time of marriage. They all craved for knowledge and felt that the right person to impart such knowledge should have been the mother or an elder sister. The challenges of transition from childhood in the parental home to married life in the new home need to be recognized at both individual and community level. Trends of modernization and globalization influence the transition from childhood to adulthood underlining the need for new ways of preparing young women for marriage. This should include information about sexuality, childbirth and inter-spousal communication acceptable to the community. Mothers will only respond to their daughters' wishes if it is socially accepted. At the same time there is need for innovative interventions to reach out to young women in disadvantaged homes. There is need to identify and provide support to such families to find suitable solutions to their difficult situation within the local context. The study shows that young women's lives are affected by the mesosystem and the exosystem of Bronfenbrenner's model. It is essential to bring rifts in the outer circles of the framework at the societal level to improve the lives of these young women entering a new phase of life.

## Competing interests

The authors declare that they have no competing interests.

## Authors' contributions

SH was the main author of the manuscript and involved in all aspects of the study. EJ and BR provided scientific oversight and feedback throughout the development of the study and manuscript. All co-authors have seen and approved the final version of the paper and have agreed to its submission for publication.

## Pre-publication history

The pre-publication history for this paper can be accessed here:


